# The Hole in the Stomach

**DOI:** 10.1155/2008/257185

**Published:** 2007-11-18

**Authors:** Hans Bödeker, Steffen Leinung, Henning Wittenburg, Julia Fischer, Ingolf Schiefke, Niels Teich

**Affiliations:** ^1^ Department of Internal Medicine II, University of Leipzig, 04103 Leipzig, Germany; ^2^ Department of Surgery II, University of Leipzig, 04103 Leipzig, Germany

## Abstract

A 57 year old woman was presented to the emergency department with upper abdominal pain and left sided chest discomfort. No cardiac or pulmonary cause could be determined and the patient underwent upper gastrointestinal endoscopy. Inversion of the scope to the fundus and subsequent fluoroscopy revealed a diaphragmatic hernia with a large herniation of the gastric fundus. Immediate laparotomy showed a 3 cm orifice of the diaphragm. The orifice was widened and a partial necrosis of the incarcerated fundus was resected. The patient recovered fully and was discharged 12 days after laparotomy.

## 1. INTRODUCTION

Diaphragmatic hernia in the adult is a rare event and most commonly occurs due to a sudden pressure gradient between the peritoneal and thoracic cavities. Rarely, no specific history can be obtained. In this report, we present the endoscopic and fluoroscopic appearance as well as the intraoperative situs of an idiopathic diaphragmatic hernia.

## 2. Case Report

**Figure 1:**
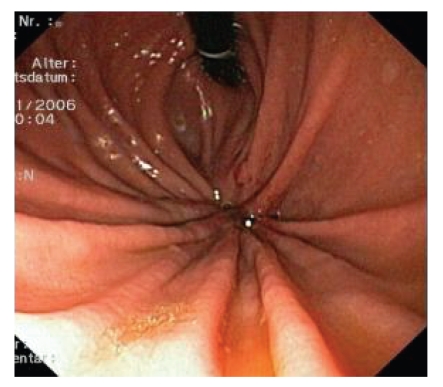
Upper gastrointestinal endoscopy: inversion view into the gastric fundus.

A 57-year-old woman (180 cm, 60 kg) was presented to the emergency department with upper abdominal pain and left-sided chest discomfort for a couple of days. In addition, she reported of nausea. Upon physical examination, percussion sounds over the left lower thorax were a bit dull and cervical lymph nodes were palpable with a size of up to 1 cm. The abdomen was generally tender without signs of peritonitis; bowel sounds were normal. The ECG showed no abnormalities. Routine laboratory tests revealed mild leucocytosis and a slightly elevated C-reactive protein level. Troponin, myoglobine, and creatine kinase levels were within normal limits. An upper gastrointestinal endoscopy was performed. Whereas the oesophagus, the lower stomach, and the duodenum were without abnormalities, a small hole in the fundus was visible after inversion of the scope (Figure 1).

**Figure 2:**
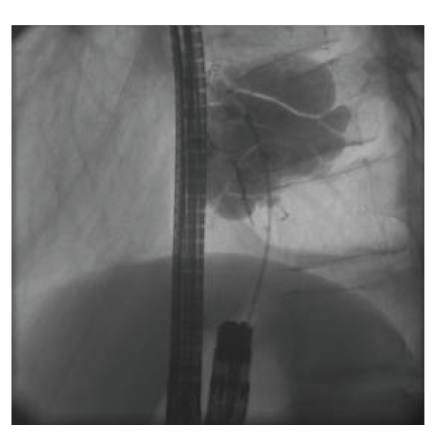
Fluoroscopy after contrast dye application via the “hole in the stomach” revealed a large intrathoracal herniation of the gastric fundus.

**Figure 3:**
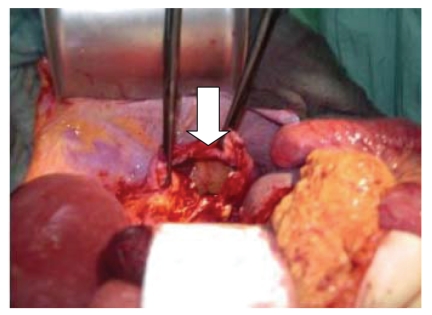
Orifice of the left part of the diaphragm (arrow).

**Figure 4:**
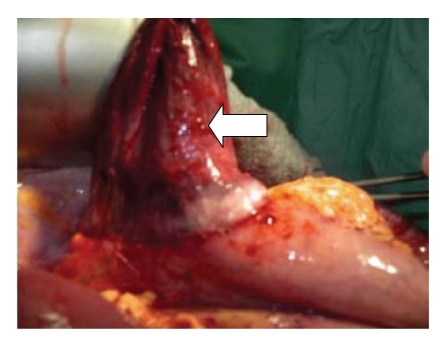
Partial necrosis of the incarcerated fundus (arrow).

The “hole in the stomach” was intubated with a catheter and contrast dye was administered. Fluoroscopy revealed a diaphragmatic hernia with a large herniation of the gastric fundus (Figure 2). Immediate laparotomy showed a 3 cm orifice of the diaphragm (Figure 3). The orifice was widened and a partial necrosis of the incarcerated fundus was resected (Figure 4). The diaphragmatic orifice was covered with polydioxanone. The patient recovered fully and was discharged 12 days after laparotomy.

## 3. Discussion

Nonhiatal diaphragmatic hernia in the adult is a rare event. Commonly, it becomes symptomatic after blunt trauma, heavy lifting, or other causes of a substantial pressure gradient between the peritoneal and thoracic cavities. Occasionally, no specific history can be obtained. The intrathoracic portion of the stomach may become strangulated and necrotic and finally perforate, resulting in the development of a hydrothorax, a gastropleural fistula, and respiratory distress [1, 2]. Therefore, in general, immediate surgery is indicated.

## References

[1] Symbas PN, Vlasis SE, Hatcher C (1986). Blunt and penetrating diaphragmatic injuries with or without herniation of organs into the chest. *The Annals of Thoracic Surgery*.

[2] Tzeng J-J, Lai K-H, Lo G-H, Hsu J-H, Mok K-T (2001). Gastropleural fistula caused by incarcerated diaphragmatic herniation of the stomach. *Gastrointestinal Endoscopy*.

